# Paroxetine combined with olanzapine in the treatment of schizophrenia

**DOI:** 10.12669/pjms.36.3.846

**Published:** 2020

**Authors:** Ning Wang, Na Zheng, Mei Dong, Jin He

**Affiliations:** 1 Ning Wang, Department of Psychiatric (III), Binzhou People’s Hospital, Shandong, 256610, China; 2 Na Zheng, Department of Psychiatric (III), Binzhou People’s Hospital, Shandong, 256610, China; 3 Mei Dong, Otolaryngology Head and Neck Surgery, Binzhou People’s Hospital, Shandong, 256610, China; 4 Jin He, Department of Psychiatric (III), Binzhou People’s Hospital, Shandong, 256610, China

**Keywords:** Depression, Olanzapine, Paroxetine, Schizophrenia

## Abstract

**Objective:**

To investigate the clinical efficacy of paroxetine combined with olanzapine in the treatment of senile schizophrenia with depression.

**Methods:**

Eighty-four elderly schizophrenic patients with depression who were admitted to our hospital from August 2016 to February 2018 were selected as research subjects and randomly divided into observation group and control group using random number table, 42 cases in each group. The control group was treated with olanzapine orally, while the observation group was treated with olanzapine and paroxetine orally. The level of homocysteine (Hcy) in the two groups was analyzed before and after treatment. The efficacy was evaluated by the Positive and Negative Symptoms Scale (PANSS) score and Hamilton Depression Scale (HAMD) score. The adverse reactions of the two groups were compared.

**Results:**

After treatment, the level of serum Hcy in the observation group was significantly lower than that in the control group (P<0.05), and it was close to the normal level. There was no significant difference in PANSS score between the two groups before treatment (P>0.05). After treatment, the negative factor score and PANSS score in the observation group were significantly lower than those in the control group, and the differences were statistically significant (P<0.05). The HAMD score of the two groups had no significant difference before treatment (P>0.05). After treatment, the HAMD score of the observation group was significantly lower than that of the control group (P<0.05). The difference of incidence of adverse reactions between the two groups had no statistical significance (P>0.05).

**Conclusion:**

Paroxetine combined with olanzapine has a definite clinical effect in the treatment of senile schizophrenia with depression. It can effectively reduce the level of serum Hcy, relieve the symptoms of schizophrenia, and alleviate the depressive symptoms of patients, with high safety. It is worth promoting.

## INTRODUCTION

Schizophrenia is one of the common psychiatric diseases in clinic. It has extremely complex pathogenesis, and its onset is slow or subacute. It has various clinical manifestations, mainly including cognition impairment and affective thinking disorder, which will severely affect the physical and psychological health of patients and their family members.^1^ It is reported that 20% ~ 70% of patients with schizophrenia will have depression symptom, which will not only affect the treatment, but also increases risks of accidents such as self-mutilation and suicide.^2^ It is generally considered that depression has been one of the most important marks of acute attack of schizophrenia.^3^ Elderly is the high-risk group of schizophrenia complicated with depression, and the clinical treatment is extremely difficult.^4^ At present, the main clinical treatment for schizophrenia complicated with depression is drug control.^5^ Although antipsychotic drugs have been available for more than 40 years, there are still about one third of schizophrenic patients who obtain poor efficacy after treatment with antipsychotic drugs. How to treat psychiatric diseases and prevent its recurrence is an important issue that puzzles psychiatric clinicians.

Whether the combination of antipsychotic drugs can be taken as a first-line recommended problem has not been recognized, but according to clinical reports and treatment guidelines, single therapy is recommended.^6^ However, in practice, in order to improve the residual positive symptoms, negative symptoms, depressive symptoms, cognitive impairment, hostile aggression symptoms or other symptoms such as obsessive-compulsive and anxious symptoms of schizophrenia and improve the efficacy of treatment of schizophrenia, combination therapy is often selected. According to the literature,^7^ 26.1% of psychiatric patients who received antipsychotic treatment in China were treated with two or more antipsychotic drugs.

Olanzapine is a second-generation antipsychotic drug which has been widely used in clinic. It can greatly reduce the occurrence of extrapyramidal symptoms in the treatment of schizophrenia. Olanzapine is a 5-hydroxytryptamine and dopamine receptor antagonist which can alleviate the positive and negative symptoms of schizophrenia and can also relieve the occurrence of bipolar depressive disorder.^8^,^9^ Paroxetine is one of the best antidepressants and anxiety drugs.^10^ It has a positive effect on improving the negative symptoms of schizophrenia patients. This study evaluated the efficacy of paroxetine combined with olanzapine in the treatment of senile schizophrenia with depression.

## METHODS

Eighty-four elderly schizophrenics with depression who were admitted to our hospital from August 2016 to February 2018 were selected as the research subjects. The inclusive criteria were: meeting the CCMD-3 diagnostic criteria for schizophrenia;^11^ age > 65 years old; positive and negative symptoms scale (PANSS) score > 60 points; and Hamilton depression scale (HAMD) score > 18 points. Exclusive criteria included having heart, lung, liver, kidney and other organ dysfunction, lactating or pregnant women, taking any antidepressant, antianxiety and other psychotropic drugs one week before admission; or being allergic or intolerant to the drugs used in this study. The 84 patients were divided into a control group and an observation group by random number table method, 42 in each group. The control group consisted of 21 males and 21 females; they aged 65-80 years, with an average age of (71.6±4.7) years, and the course of disease ranged from 2 to 12 years (average (7.7±2.6) years). In the observation group, there were 27 males and 22 females; they aged 65-82 years, with an average age of (71.3±4.5) years, and the course of disease was 2-11 years (average (7.3±2.8) years). There was no significant difference in general data between the two groups; thus the results were comparable. All patients gave informed consent and the study protocol has been approved by the ethics committee of the hospital.

### Therapeutic methods

The control group was treated with olanzapine tablets (Jiangsu Hansoh Pharmaceutical Co., Ltd., China; Approval Number: H20010799; specification: 10 mg/tablet). The initial dosage was one tablet per day (10 mg). The clinical symptoms were evaluated, and patients with severe condition were given two tablets per day (20 mg) and given one tablet per day after the disease condition were stabilized.

On the basis of taking olanzapine as the control group, patients in the observation group orally took paroxetine hydrochloride tablets (Zhejiang Jianfeng Pharmaceutical Co., Ltd., China; approval number: H20040533; specifications: 20 mg * 12 tablets) at a draught at breakfast every day. The tablets were swallowed without chewing, one tablet per day. Both groups were treated for eight weeks.

During treatment, all patients cooperated with the following nursing cares. During the acute treatment period, the nurses arranged the patients in the key wards within 24 hours after admission, carried out relevant examinations in the perspective of their specific conditions, strictly supervised their medication situation; carefully observed the changes of patients’ mental state after taking medication, and provided appropriate life guidance according to the actual situation of patients, including helping them to keep good health habits and give them relevant guidance to ensure their adequate sleep. According to the needs of patients, the nurses carried out a detailed analysis of their psychological state and evaluated their psychological states along with the patients. Personalized psychological counseling was implemented according to the evaluation results. The next stage of nursing started when the patients’ condition was significantly improved, their life self-care ability basically restored, and they had a clear awareness of their illness and have good sleep. In the rehabilitation treatment period, the patients were forbidden from going out. If they must go out, special person was designated to accompany them. The continuous nursing in the last step was continued for 24 hours, and their psychological intervention was enhanced. Patients who had hallucinations or delusions were encouraged to talk about topics in which they were interested and distracted by watching TV, playing cards, playing chess, playing mahjong and other methods. The patients were allowed to go to rehabilitation centers for treatment. Their interpersonal skills, life skills and drug self-management skills were enhanced through lectures and multimedia. More encouragement and care were given to the patients to improve their compliance with medication. Targeted psychological counseling was implemented according to their emotional and psychological states. The next stage of nursing care started when the patients had a correct understanding of their own diseases, could take food and medicine on time and actively, and could do rehabilitation training actively.

In the period of consolidation treatment, the patients were arranged in general wards and were allowed to go out. Their medication and diet conditions were checked and monitored, and bad behaviors were corrected. Mental health knowledge education was enhanced. They were guided to overcome their own personality weaknesses, so as to actively face their own diseases and enhance confidence in future life. They were instructed to strictly comply with medical advice after discharge and regularly returned to the hospital for review. They were encouraged to actively participate in healthy collective activities. When the medication condition, daily life and interpersonal contacts returned to normal and they had a correct understanding of their diseases, they were allowed to discharge.

### Observation indicators

Before and after treatment, two mL of fasting elbow and wrist venous blood was extracted and serum Hcy level was detected by high performance liquid chromatography. 5-16 μmol/L was considered as the normal level.

PANSS was used before and after treatment.^12^ The scale consisted of seven negative symptoms, seven positive symptoms and 16 general psychopathological symptoms. Each item was scored by 7-grade scoring method. No symptoms, very mild symptoms, mild symptoms, moderate symptoms, slightly severe symptoms, severe symptoms and extremely severe symptoms were scored 1~7 points respectively. The lower the score, the lighter the symptoms.

HAMD scale was also used before and after treatment. The scale applied 5-grade scoring method. No symptoms, mild symptoms, moderate symptoms, severe symptoms and extremely severe symptoms were given 0-4 points respectively. The total score ≥ 35 was evaluated as severe depression. The lower the score, the lighter the degree of depression. The adverse reactions of the two groups after medication were observed and compared.

### Statistical analysis

SPSS 21.0 was used for data processing. The measurement data were expressed as Mean±SD, and the comparison between groups applied t test. The counting data was expressed as percentage, and the comparison between groups applied X^2^ test. The difference was statistically significant if P<0.05.

## RESULTS

Before treatment, there was no significant difference in the level of Hcy between the two groups (P>0.05), and it was higher than normal. After treatment, the level of Hcy in the two groups decreased significantly, and the level of serum Hcy in the observation group was lower than that in the control group, and close to the normal level; there was a significant difference between the two groups (P<0.05, Table-I).

**Table-I:** Serum Hcy level between two groups before and after treatment (μmol/L).

Group	Observation group	Control group
Before treatment	24.77±5.61	25.24±5.48
After treatment	12.07±2.38*^#^	17.36±2.75*

***Note:***

* meant that P<0.05 compared to before treatment;

# meant that P<0.05 compared to the control group.

Before treatment, there was no significant difference in the scores of negative factors, positive factors and general psychopathology between the two groups (P>0.05). After treatment, there was no significant difference in the scores of positive factors and general psychopathology between the two groups (P>0.05); the score of negative factors and the total score in the observation group were lower than those in the control group, and the difference was significant (P<0.05, Table-II.

**Table-II:** PANSS score between the two groups before and after treatment (Mean±SD).

Group		Observation group	Control group
Negative factor score	Before treatment	35.2±4.8	35.5±5.2
	After treatment	25.7±4.2*^#^	32.2±4.1*
Positive factor score	Before treatment	14.8±3.6	14.7±3.7
	After treatment	11.1±2.3*	11.3±2.2*
General psychopathological score	Before treatment	24.6±3.8	24.8±3.7
	After treatment	20.3±3.4*	21.5±3.2*
Total score	Before treatment	73.7±11.6	74.3±10.2
	After treatment	57.2±8.6*^#^	64.7±7.6*

***Note:***

* meant that P<0.05 compared to before treatment;

# meant that P<0.05 compared to the control group.

Before treatment, there was no significant difference in the HAMD score between the two groups (P>0.05); after treatment, the HAMD score of the two groups was significantly different. The score of HAMD in the observation group was significantly lower than that in the control group after treatment (P<0.05, Table-III).

**Table-III:** HAMD score between two groups before and after treatment.

Group	Observation group	Control group
Before treatment	25.36±3.64	25.66±3.45
After treatment	10.50±2.45*^#^	20.16±2.48*

No adverse reactions such as anorexiam diarrhea, insomnia, loss of appetite, occasional angioedema, urticaria, postural hypotension and extrapyramidal reactions occurred in the two groups. In the control group, there were three cases of headache, tjree cases of nausea and vomiting, and five cases of fatigue, and the incidence of adverse reactions was 26.2%. In the observation group, there were 4 cases of headache, four cases of nausea and vomiting, and 6 cases of fatigue, and the incidence of adverse reactions was 33.3%. There was no significant difference in the incidence of adverse reactions between the two groups (X^2^=0.333, P>0.05).

## DISCUSSION

In recent years, the incidence of schizophrenia has gradually increased, and the number of elderly patients shows an upward trend. Once suffering from schizophrenia, it is very easy to have depression.^13^ According to statistics, more than 60% of patients with the first-episode schizophrenia will have depression, and about half of the recurrent patients are possible to have depression.^14^ For the elderly patients, the incidence of depression is relatively higher. Moreover, the elderly patients are more sensitive to clinical adverse reactions of anti-psychiatric and depressive drugs because of the decrease of metabolic function.^15^ Therefore; it is of positive significance to strengthen research on the clinical treatment of elderly patients with schizophrenia and depression.

Olanzapine, a triphenyl diazepine antipsychotic drug, can significantly block dopamine receptors and serotonin receptors in the human brain. It is a typical drug for the treatment of schizophrenia.^16^ Schizophrenia has positive and negative symptoms. Olanzapine is mainly used in the treatment of mental illness with positive symptoms and the maintenance treatment of secondary emotional symptoms in the acute phase. It has a weak effect on mental illness with negative symptoms.^17^ Guo et al. found that olanzapine combined with escitalopram was better than olanzapine alone in the treatment of schizophrenia with depression.^18^ Paroxetine is mostly used to treat depression patients with anxiety disorder. It has good affinity with 5-HT receptor and can selectively inhibit the re-uptake of 5-HT, effectively block the recovery of 5-HT, and combine with olanzapine to make up for its weak effect on schizophrenia with negative symptoms.^19^ Olgiati et al. has reported that paroxetine had significant effect in the treatment of geriatric depression.^20^ The results of the present study showed that the scores of negative factor, PANSS and HAMD of the observation group decreased after treatment and were significantly better than those of the control group, indicating that olanzapine combined with paroxetine could effectively improve the therapeutic effect in the treatment of schizophrenia and play a coordinated role, which was similar to the results of Reavley et al.^21^ Moreover the observation of adverse reactions demonstrated that there was no significant difference in the adverse reactions between the two groups, suggesting that the combined use of drugs had no influence on the safety.

Recent studies have shown that the occurrence of schizophrenia is closely related to the abnormal metabolism of Hcy.^22^,^23^ Hcy is an intermediate product that can enter the nervous center through the blood-brain barrier and is produced by methionine methylation. It can mediate the production of choline, 5-HT, DA and other neurotransmitters, induce neuronal apoptosis, and inhibit the expression of various anti-apoptotic proteins to produce neurotoxic effects and aggravate oxidative stress reaction, thereby causing diseases such as schizophrenia. The results of this study showed that paroxetine combined with olanzapine reduced serum Hcy to normal level, and although olanzapine alone decreased the serum Hcy level, it was still higher than normal level.

## CONCLUSION

Paroxetine combined with olanzapine has a synergistic effect, which can effectively reduce serum Hcy to normal level, improve patients’ mental symptoms and alleviate depressive symptoms. Moreover, paroxetine combined with olanzapine is a useful and effective drug combination for the treatment of schizophrenia. It is worth promoting in clinical practice. However, due to the small sample size and short observation time in this study, some adverse drug reactions might not be fully manifested and need further observation in the future.

### Authors’ Contribution:

**NW & NZ:** Study design, data collection and analysis. **NZ, MD & JH:** Manuscript preparation, drafting and revising.**NW& JH:** Review and final approval of manuscript, are responsible for integrity of research.
